# Acquisition of *FGFR1* and *NSD3* Amplifications During the Transformation of *EGFR*-Mutated Lung Adenocarcinoma into Squamous Cell Carcinoma: A Case Report

**DOI:** 10.1016/j.jtocrr.2025.100862

**Published:** 2025-06-13

**Authors:** Naoki Fukunaga, Hideki Terai, Rui Nomura, Yutaka Kurebayashi, Kohei Nakamura, Ryutaro Kawano, Kohei Shigeta, Koji Okabayashi, Katsuhito Kinoshita, Akihiko Ogata, Lisa Shigematsu, Fumimaro Ito, Hatsuyo Takaoka, Takahiro Fukushima, Shigenari Nukaga, Keiko Ohgino, Hiroyuki Yasuda, Hiroshi Nishihara, Yuko Kitagawa, Koichi Fukunaga

**Affiliations:** a Division of Pulmonary Medicine, Department of Medicine, Keio University School of Medicine, Tokyo, Japan; b Keio Cancer Center, Department of Medicine, Keio University School of Medicine, Tokyo, Japan; c Department of Pathology, Keio University School of Medicine, Tokyo, Japan; d Center for Cancer Genomics, Keio University School of Medicine, Tokyo, Japan; e Department of Surgery, Keio University School of Medicine, Tokyo, Japan; f Division of Pulmonary Medicine, Saitama Municipal Hospital, Saitama, Japan

**Keywords:** EGFR mutation–positive lung adenocarcinoma, Squamous cell transformation, *NSD3* amplification, *FGFR1* amplification, Case report

## Abstract

Histologic transformation from adenocarcinoma to SCLC is a recognized mechanism of resistance in lung cancer. However, the transformation into squamous cell carcinoma is less common, and the associated genomic alterations remain unclear. Here, we present a case of lung adenocarcinoma harboring an EGFR (*EGFR*) mutation that transformed into squamous cell carcinoma. Although *EGFR* L858R mutation was detected throughout the transformation, genomic analyses were performed during the disease course, revealing the amplification of *FGFR1* and *NSD3*, which have recently been proposed as potential driver oncogenes in lung squamous cell carcinoma. This case report highlights the genomic alterations observed in repeatedly biopsied specimens, along with a review of the relevant literature.

## Introduction

*EGFR* mutations are key drivers of lung adenocarcinoma, particularly in East Asian populations. Although EGFR-tyrosine kinase inhibitors (TKIs) are initially effective, resistance typically emerges within 1 to 2 years because of various mechanisms.^1^ Among these, histologic transformation is a well-recognized resistance mechanism to EGFR-TKIs. Transformation to SCLC is well-known and most frequent (67%),^2^ whereas transformation to squamous cell lung carcinoma has also been reported, although less frequently (15%), and remains poorly understood.^2^ In this report, we describe a case in which multiple clinical specimens were obtained over the course of the disease, allowing us to detect genomic alterations before and after the transformation. Notably, we identified *FGFR1* and *NSD3* amplifications, which have recently been proposed as potential driver oncogenes in lung squamous cell carcinoma.^3^ These findings, alongside tumor characteristics, offer insights into the transformation of lung adenocarcinoma into lung squamous cell carcinoma.

## Case Presentation

**Figure 1:**
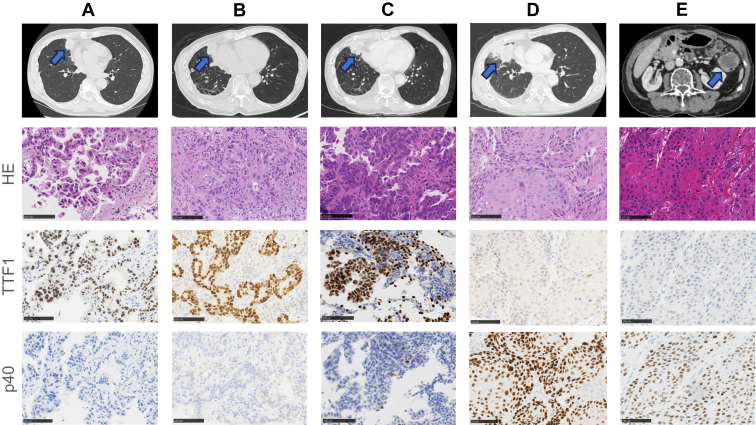
The biopsy sites in computed tomography scans and the corresponding histologic findings (Scale bars: 100 μm). (*A*) Histologic staining (H&E) of the first biopsy performed at the primary lesion confirmed adenocarcinoma. Immunostaining revealed nuclear positivity for TTF1 and negativity for p40, supporting the diagnosis of adenocarcinoma. (*B*) Immunostaining of the second biopsy also revealed nuclear positivity for TTF1 and negativity for p40, consistent with adenocarcinoma. (*C*) H&E staining of the third biopsy revealed a retained glandular structure, consistent with adenocarcinoma, including immunostaining. (*D*) H&E staining of the fourth biopsy was negative for nuclear TTF1 and positive for nuclear p40, indicative of squamous cell carcinoma. (*E*) H&E staining of the resected intestinal specimen indicated squamous cell carcinoma with well-defined keratinization. Immunostaining revealed negative TTF1 and positive p40, confirming squamous cell carcinoma. H&E, hematoxylin and eosin.

**Figure 2:**
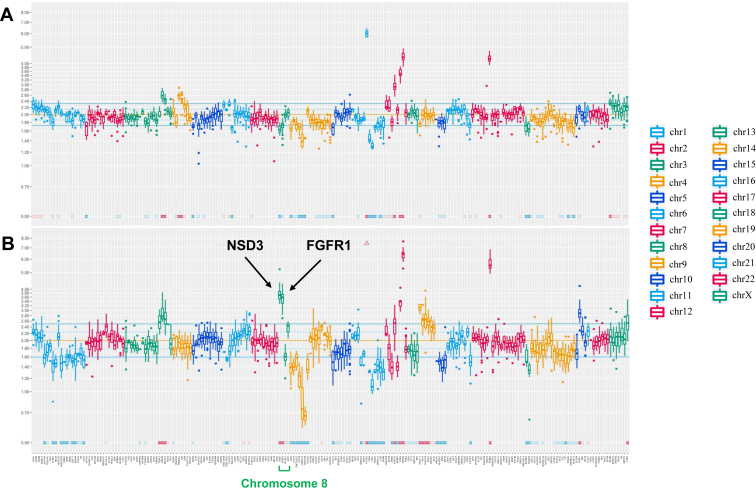
Comparison of copy number variations before (*A*) (third biopsy) and after (*B*) (resected metastasis) histologic transformation. An increase in the copy numbers of *NSD3* and *FGFR1* on chromosome 8 is observed after transformation. Chromosomal regions are color-coded as indicated.

A 66-year-old man with no marked medical or family history and a previous 8-pack-year history presented in June 2018 with a cough. A computed tomography (CT) scan revealed a tumor in the right middle lobe, and a CT-guided biopsy confirmed a diagnosis of lung adenocarcinoma with pleural dissemination. The tumor was staged as cT3N0M1a (stage IVa), and an EGFR L858R mutation was identified using the Cobas *EGFR* mutation test (Fig. 1*A*). First-line treatment with afatinib was initiated, leading to a reduction in the primary tumor size and a partial response. In June 2021, after the enlargement of the primary tumor, a second CT-guided biopsy was performed at the primary tumor site. Pathologic analysis confirmed lung adenocarcinoma (Fig. 1*B*), and the Cobas *EGFR* mutation test detected both the L858R and T790M mutations. Consequently, second-line treatment with osimertinib was started. Approximately 10 months after, the primary tumor exhibited further enlargement, indicating progressive disease. In March 2022, a third CT-guided biopsy at the same site and histologic examination revealed adenocarcinoma with increased atypia (Fig. 1*C*). The third biopsy sample was submitted to Rapid-Neo, a next-generation sequencing system for clinical specimens establised with a SureSelect custom-designed panel (Agilent Technologies).^4^ The sample tested positive for *EGFR* L858R mutation but was negative for T790M. Subsequently, the patient was started on third-line therapy, including carboplatin, pemetrexed, and bevacizumab. A tumor assessment in November 2022 indicated progressive disease, as evidenced by an increase in the size of the primary lesion, and the treatment was changed to docetaxel and ramucirumab as the fourth-line therapy. Given the rapid growth of the primary lesion during the fourth-line treatment, we suspected histologic tumor transformation. In March 2023, a repeat biopsy by bronchoscopy confirmed the transformation to squamous cell carcinoma (Fig. 1*D*). The tissue was submitted to FoundationOne (Foundation Medicine, Inc., Cambridge, MA), which revealed *NSD3* and *FGFR1* amplification, findings that had not been detected in the previous next-generation sequencing analysis using Rapid-Neo (Table 1). These alterations were not assessed in the first and second biopsy samples, which underwent only polymerase chain reaction–based *EGFR* testing. Radiotherapy (60 Gy/30 fractions) was administered to the primary tumor, followed by fifth-line therapy with carboplatin and S-1. However, after approximately 2 months, new metastatic lesions were detected in the posterior neck subcutaneous area, left axillary lymph node, and descending colon, although the previously identified metastatic lesions remained confined to the pleural cavity. In September 2023, the tumor in the descending colon was surgically removed because of the risk of perforation. Pathologic analysis confirmed squamous cell carcinoma, diagnosed as metastatic lung cancer (Fig. 1*E*). The tissue was submitted to the Rapid-Neo, which retained amplification of *NSD3* and *FGFR1* (Fig. 2*A* and *B*), although these amplifications were not detected in the third biopsy. In October 2023, gemcitabine was initiated as the sixth-line treatment, followed by afatinib rechallenge and vinorelbine as the seventh and eighth lines, respectively. However, tumor progression could not be controlled, and the patient died in July 2024.

**Table 1:** Genetic Alterations Detected at Each Examination

Alteration Type	Genetic Alterations	Primary Lung (ADC)	Primary Lung (ADC)	Primary Lung (ADC)	Primary Lung (SCC)	Metastatic Colon (SCC)
		EGFR-PCR (Cobas)	EGFR-PCR (Cobas)	CGP (Rapid Neo)	CGP (FoundationOne)	CGP (Rapid Neo)
		2018.07	2021.06	2022.03	2023.03	2023.09
Mutation	*EGFR* p.L858R	Detected	Detected	Detected	Detected	Detected
	*EGFR* p.T790M	Not detected	Detected	Not detected	Not detected	Not detected
	*PIK3CA* p.E545K	Not assessed	Not assessed	Detected	Detected	Detected
	*RET* p.V804M	Not assessed	Not assessed	Detected	Detected	Detected
	*RB1* c.1815-1G>A	Not assessed	Not assessed	Detected	Detected	Detected
	*RBM10* p.G465fs^∗^20	Not assessed	Not assessed	Detected	Detected	Detected
Copy number alteration	*CCND1*	Not assessed	Not assessed	Amplification	Amplification	Amplification
	*MDM2*	Not assessed	Not assessed	Amplification	Amplification	Amplification
	*ERBB2*	Not assessed	Not assessed	Amplification	Amplification	Amplification
	*CDK4*	Not assessed	Not assessed	Amplification	Neutral	Amplification
	*FGFR1*	Not assessed	Not assessed	Neutral	Amplification	Amplification
	*NSD3 (WHSC1L1)*	Not assessed	Not assessed	Neutral	Amplification	Amplification
	*CDKN2A*	Not assessed	Not assessed	Homozygous deletion	Homozygous deletion	Homozygous deletion
	*CDKN2B*	Not assessed	Not assessed	Homozygous deletion	Homozygous deletion	Homozygous deletion

ADC, adenocarcinoma; CGP, comprehensive genomic profiling; PCR, polymerase chain reaction; SCC, squamous cell carcinoma.

The autopsy revealed that the primary lesion was predominantly composed of squamous cell carcinoma; however, a minor adenocarcinoma component was also identified. Although most metastases were squamous cell carcinoma and were found outside the thorax, most metastases of adenocarcinoma were localized within the thoracic cavity.

## Discussion

Compared with the transformation of lung adenocarcinoma to small-cell carcinoma, reports on the genomic information before and after transformation to squamous cell carcinoma remain limited (Supplementary Table 1). Alterations in the PI3K/AKT/mTOR pathway and, more recently, *NSD3* amplification have been identified as major drivers of transformation to squamous cell carcinoma.^3^ Repeated genomic testing poses substantial challenges in clinical practice. To the best of our knowledge, this is the first case report identifying *FGFR1* and *NSD3* amplification exclusively in a posttransformation specimen, underscoring their potential roles in the transformation to squamous cell lung cancer. The influence of multiple lines of therapy on the tumor microenvironment should also be considered, as treatment pressures may have contributed to the observed genomic and histologic changes, potentially driving the transformation process. Moreover, the persistent detection of the *EGFR* L858R mutation suggests that this case represents histologic transformation rather than de novo squamous cell carcinoma. A limitation of this case study is that different genomic profiling platforms were used for each biopsy, because of constraints related to insurance coverage and sample quality. Furthermore, the initial biopsy sample did not undergo broad-panel genomic testing, which may have precluded early detection of relevant genomic alterations.

Accumulating such cases will clarify squamous cell carcinoma transformation mechanisms and improve personalized lung cancer treatments.

In conclusion, we report a case of *EGFR* mutation–positive lung adenocarcinoma that transformed into squamous cell carcinoma. Repeated biopsies and sequencing revealed intratumor genomic and histologic alterations that potentially led to the transformation. Sequencing at the time of rebiopsy provides valuable insights into the mechanisms underlying transformation and cancer progression.

## CRediT Authorship Contribution Statement

**Naoki Fukunaga:** Investigation, Writing - original draft.

**Hideki Terai:** Conceptualization, Supervision, Writing - review & editing.

**Rui Nomura:** Writing - review & editing.

**Yutaka Kurebayashi:** Writing - review & editing.

**Kohei Nakamura:** Writing - review & editing.

**Ryutaro Kawano:** Writing - review & editing.

**Kohei Shigeta:** Writing - review & editing.

**Koji Okabayashi:** Writing - review & editing.

**Katsuhito Kinoshita:** Writing - review & editing.

**Akihiko Ogata:** Writing - review & editing.

**Lisa Shigematsu:** Writing - review & editing.

**Fumimaro Ito:** Writing - review & editing.

**Hatsuyo Takaoka:** Writing - review & editing.

**Takahiro Fukushima:** Writing - review & editing.

**Shigenari Nukaga:** Writing - review & editing.

**Keiko Ohgino:** Writing - review & editing.

**Hiroyuki Yasuda:** Writing - review & editing.

**Hiroshi Nishihara:** Writing - review & editing.

**Yuko Kitagawa:** Writing - review & editing.

**Koichi Fukunaga**: Writing - review & editing.

## Disclosure

The authors declare no conflict of interest.

## References

[1] Leonetti A., Sharma S., Minari R., Perego P., Giovannetti E., Tiseo M. (2019). Resistance mechanisms to osimertinib in EGFR-mutated non-small cell lung cancer. Br J Cancer.

[2] Fujimoto D., Akamatsu H., Morimoto T. (2022). Histologic transformation of epidermal growth factor receptor-mutated lung cancer. Eur J Cancer.

[3] Yuan G., Flores N.M., Hausmann S. (2021). Elevated NSD3 histone methylation activity drives squamous cell lung cancer. Nature.

[4] Nukaya T., Sumitomo M., Sugihara E. (2023). Estimating copy number to determine BRCA2 deletion status and to expect prognosis in localized prostate cancer. Cancer Med.

