# Supplementary material for: Acquisition of FGFR1 and NSD3 Amplifications During the Transformation of EGFR-Mutated Lung Adenocarcinoma into Squamous Cell Carcinoma: A Case Report
Source: JTO Clin Res Rep. 2025 Jun 13;6(9):100862. doi: 10.1016/j.jtocrr.2025.100862 (PMC12335958; doi:10.1016/j.jtocrr.2025.100862)
Supplement: Supplementary Table 1 [file mmc1.pdf]

Table2. Characteristics of patients with genomic imformation transformation to SCC from primary lung ADC after EGFR-TKIs treatment.

References	Gender	Age	Smoking	EGFR mutations before resistance	Genetic alterations other than the EGFR gene before resistance	Initial TKI	Duration of response to EGFR-TKI (months)	EGFR mutations after resistance	Genetic alterations other than the EGFR gene after resistance	Treatment regimen after histologic transformation	Survival period after histologic transformation (months)
1 Kuiper et al.	F	63	N	ex21(L858R)	NA	Gefitinib	5	None	PIK3CA	Gefitinib	6
2 Park et al.	F	65	N	ex21(L858R)	CTNNB1, TP53, IDH2, GRIN2A	Gefitinib	15.6	None	ROBO2, PTPRD, MED12, PTEN(1n loss), PTEN, CDH5, CHEK2	Carboplatin + Paclitaxel	29.5
2 Park et al.	M	56	Y	ex19(del)	LRP1B, PIK3CA	Afatinib	7.1	None	PTEN	Everolimus	15.5
2 Park et al.	M	40	Y	ex19(del)	LTK, TSC1, TP53, PAX5(amp), INPP4B(amp), EPHB1, RB1, FGF7	Afatinib	27.6	T790M	PTEN(1n loss), CDKN2A/2B(del), FANCF(del), HRAS(del), LMO1(del), DOCK2, CHD4, TP53, GNAS	Osimertinib	46.1
2 Park et al.	M	67	Y	ex19(del)	CCNE1, PIK3CA, CCNE1(amp), PMS2(amp), HOXA(amp), FANCA, GNAQ, MLH1, NF1, PHLPP2, WHSC1L1, PIK3CG, BRAF	Erlotinib	29.8	T790M	RAD51B, RICTOR	Osimertinib	32.5
3 Uruga et al.	M	61	Y	ex19(ins)	NA	Erlotinib	28	T790M	PTEN (loss), PDGFRA, HRAS	Osimertinib	17
3 Uruga et al.	M	72	Y	ex21(L858R)	NA	Erlotinib	9	T790M	PTEN (loss), TP53, SMARCB1, KIT	Osimertinib	8
4 Chiang et al.	F	54	N	ex21(H835L,L833V) ex19(T790M)	NA	Gefitinib	19	None	TP53, mTOR	Osimertinib + Everolimus	3*
5 Schoenfeld et al.	NR	NR	NR	NR	TP53, 3q gain	Osimertinib	NR	NR	ND	Gemcitabine + Vinorelbine	10
5 Schoenfeld et al.	NR	NR	NR	NR	ND	Osimertinib	NR	NR	PIK3CA, BRCA2	Pembrolizumab	4
5 Schoenfeld et al.	NR	NR	NR	NR	TP53, RB1, SMARCA4, FGFR2	Osimertinib	NR	NR	ND	Carboplatin + Etoposide	9
5 Schoenfeld et al.	NR	NR	NR	NR	TP53	Osimertinib	NR	NR	ND	Carboplatin + nab-Paclitaxel + Pembrolizumab	2
5 Schoenfeld et al.	NR	NR	NR	NR	NF1/NF2	Osimertinib	NR	NR	SMARCA4	Carboplatin + nab-Paclitaxel + Pembrolizumab	4
6 Bruno et al.	M	38	Y	ex21(L858R)	NA	Erlotinib	15	T790M	MET (amp), TP3	Osimertinib	6
7 Xi et al.	M	59	N	ex19(del)	NA	Icotinib	5	T790M	MET (amp)	Carboplatin + Paclitaxel + Pembrolizumab	20

NA = Not Assessed: parameters not evaluated or measured, ND = Not Detected: parameters measured but below detectable limits, NR = Not Reported: information not provided in the original publication, *censoring during the last follow-up.

1 Kuiper JL, Ronden MI, Becker A, Heideman DA, van Hengel P, Ylstra B, et al. Transformation to a squamous cell carcinoma phenotype of an EGFR-mutated NSCLC patient after treatment with an EGFR-tyrosine kinase inhibitor. J Clin Pathol. 2015; 68:320–1. doi: 10.1136/jclinpath-2015-202866.

2 Park S, Shim JH, Lee B, Cho I, Park WY, Kim Y, et al. Paired genomic analysis of squamous cell carcinoma transformed from EGFR-mutated lung adenocarcinoma. Lung Cancer. 2019; 134:7–15. doi: 10.1016/j.lungcan.2019.05.024.

3 Uruga H, Fujii T, Nakamura N, Moriguchi S, Kishi K, Takaya H. Squamous cell transformation as a mechanism of acquired resistance to tyrosine kinase inhibitor in EGFR-mutated lung adenocarcinoma: a report of two cases. Respirol Case Rep. 2020; 8:e00521. doi: 10.1002/rcr2.521.

4 Chiang CL, Yeh YC, Chou TY, Chiu CH. Squamous cell carcinoma transformation after acquired resistance to osimertinib in a patient with lung adenocarcinoma harboring uncommon EGFR mutation. J Formos Med Assoc. 2020; 119:1439–41. doi: 10.1016/j.jfma.2019.12.017.

5 Schoenfeld AJ, Chan JM, Kubota D, Sato H, Rizvi H, Daneshbod Y, et al. Tumor analyses reveal squamous transformation and off-target alterations as early resistance mechanisms to first-line osimertinib in EGFR-mutant lung cancer. Clin Cancer Res. 2020; 26:2654–63. doi: 10.1158/1078-0432.CCR-19-3563.

6 Bruno R, Del Re M, Cucchiara F, et al. Multiple resistance mechanisms to tyrosine kinase inhibitors in EGFR-mutated lung adenocarcinoma: a case report harboring EGFR mutations, MET amplification, and squamous cell transformation. Front Oncol. 2021; 11:674604. doi: 10.3389/fonc.2021.674604.

7 Xi YZ, Xie L, Tan XW, Zeng SL. Transformation of adenocarcinoma to squamous cell carcinoma as a source of EGFR-TKI resistance: a case report and literature review. Front Oncol. 2022; 12:942084. doi: 10.3389/fonc.2022.942084.
